# Supplementary material for: Policy driven changes in animal research practices: mapping researchers’ attitudes towards animal-free innovations using the Netherlands as an example
Source: Res Integr Peer Rev. 2019 Apr 23;4:8. doi: 10.1186/s41073-019-0067-5 (PMC6477718; doi:10.1186/s41073-019-0067-5)
Supplement: Supplementary file 1 — Supplementary material. (DOCX 65 kb) [file 41073_2019_67_MOESM1_ESM.docx]

**S1 Text. Full table of demographics**

**Table S1A. Level of responders working in work field related to animal experiments.**

| Level | N |
| --- | --- |
| PhD and MSc | 139 |
| Principle investigator | 92 |
| Post-doc | 58 |
| Head of the department | 26 |
| Regular employee | 15 |
| Analyst and biotechnician | 11 |
| Director | 11 |
| Other | 15 |
| Total | 367 |

**Table S1B. Age category of responders**

| Age category | N |
| --- | --- |
| 20-29 | 92 |
| 30-39 | 110 |
| 40-49 | 58 |
| 50-59 | 54 |
| 60+ | 16 |
| Missing | 37 |

**Table S1C. Nationality of responders**

| Inside or outside European Union | Nationality | N |
| --- | --- | --- |
| European Union | Dutch | 209 |
|  | German | 45 |
|  | French | 11 |
|  | Italian | 7 |
|  | Spanish | 7 |
|  | Portuguese | 5 |
|  | Belgian | 3 |
|  | Greek | 2 |
|  | Romanian | 2 |
|  | Serbian | 1 |
|  | Swedish | 1 |
|  | Austria | 1 |
|  | Slovene | 1 |
| Outside European Union | American | 7 |
|  | Canadian | 7 |
|  | Swiss | 5 |
|  | Chinese | 3 |
|  | Australian | 2 |
|  | British | 2 |
|  | Indian | 2 |
|  | Iranian | 1 |
|  | Korean | 1 |
|  | Russian | 1 |
|  | Thai | 1 |
|  | Turkish | 1 |
|  | Missing | 39 |

**Table S1C. Responders working in native country or abroad.**

| Working in native country or abroad | N |
| --- | --- |
| Native country | 261 |
| Abroad | 64 |
| Missing | 42 |

**S2 Text. Description of the roadblocks**

Alternatives are not animal-free

By using *in vitro* and *in silico* techniques, the complexity of the model system can be reduced. This can be done, because it causes replacing or reducing of the use of animals in experiments. Therefore, these models can be seen as alternatives. However, one cannot assume that these alternatives are necessarily animal-free. For example, the use of culture medium including bovine serum or monoclonal antibodies is regarded an alternative method without being animal-free [1-3].

Awareness is lacking

The lack of open access of data, sharing both positive and negative results, and openness in sharing thoughts and experiences, will contribute to repetition and unnecessary experimentation in directions that might not be helpful to find the answer to the stated hypothesis [4, 5]. Therefore, the awareness of already established models or performed experiments is lacking, which accounts for both animal experiments and alternatives for animal experiments.

Costs of implementation

In order to implement new models, one has to invest in the development of these new (alternative) models. Additionally, the risk that developers of an alternative take can be a hurdle when deciding to start developing such a method. The risk can be seen as an important factor in the total costs, whereas high risks might withhold developers to invest due to costly attempts.

Differences in regulation

With the implementation of the Directive 2010/63/EU, animal experimentation became regulated at a European level. However, the right to make additional national laws regarding animal experimentation remained. The legislation around animal experimentation within each country is mainly based on the possibilities that a country has to conduct science and develop itself.

Ethical issues

Alternative models for animal experimentation can face ethical issues, despite the fact that they do not directly involve animal or human experimentation. This is because some alternative methods still rely on data from animal studies in order to be developed. So, in creating solutions for using fewer animals for experiments themselves, more animals are sometimes needed for other aspects.

Pressure to conform

Science is conducted under influence of numerous laws and has a strong hierarchical personnel structure. Furthermore, the production of science should occur along strict lines to ensure the independency and reproducibility of studies, and thereby ensure the integrity of science [6]. Besides having official laws and guidelines, the diversity of the scientific community, including journals, creates its own habits and an own way of conducting science.

Publishing in high-impact journals

Journals have their own guidelines that allow researchers to publish in their journals. In most cases, it is easier to publish new data if the methods or models that are used, already have been used – and therefore are accepted as a good experimental model – in earlier publications. Additionally, reviewers play an important role in the decision which articles are accepted. Besides that, most journals are reluctant to publish negative results despite their valuable results for the general understanding of a hypothesis.

Reliability

For both animal testing and alternatives, the scientific validation and reliability are probably the most important parts to complete. Besides that, the methods used for conducting science need to be relevant and must be reproducible [7]. In order to establish this *in vivo* data with sufficient quality is needed, also to evaluate the results that are obtained in *in vitro* models. However, even if good animal-free methods are present, the comparison with animal data seems to be the key player, since animal testing is seen as the *gold standard* by a majority of the scientific community. Translation from a model – whether animal or alternative – to humans will always remain an uncertainty because a model is never 100% comparable. Therefore, the reliability of data obtained from a model can be questioned once translated to human subjects.

Research funding

Most academic research depends on money derived from funding agents, grants and awards. This funding is often awarded based on the quality and innovativeness of the research. For the development of alternative models for animal models, researchers can apply and rely on funding from the funding agencies. However, for the validation process this support is often missing [8, 9]. In this manner, funding has a major impact on both the performance of alternative research as well as the development of alternative techniques.

Time/effort to develop alternatives

When a research institution is familiar with the process of obtaining animal models it will take time and effort to deviate from this process. New information has to be gathered, expertise has to be acquired as well as the production, and validation of the newly established alternative method will take additional time and resources, compared to the use of already established animal models.

**S3 Text. Structure of the survey**

Types of questions that were used and how they are displayed in this overview:

1. Topic

1.1 Question

- Multiple choice, only one answer can be given.

1.2 Open question

1.3 Question? * (this question is obligatory)

1.4 Question

- Multiple choice, multiple answers can be given.

## S3.1: Introduction text

Dear participant,

First of all thank you for opening the link and taking time to fill in this quick survey about animal research and animal-free innovations. It will take you less than five minutes. We are a group of master’s students and we are participating in the interdisciplinary honours programme of the Radboud University Nijmegen. During this programme, we are investigating the use of alternative methods and roadblocks that may play a role in using them. All responses will be completely anonymous – no identifying personal information will be asked for or collected and you can opt out at any time.

Thank you in advance for filling in this survey!

## S3.2: National goal

The Dutch government wants to be the world leading country in animal-free innovations by 2025 (<http://bit.ly/Government2025>).

1. What do you think of the number of animals that is currently used in your field? *

- Excessive
- Sufficient
- Insufficient
- No animals are used in my field

1. Please explain your answer:
2. Do you think that research should be animal free? *

- Yes
- No
- I don’t know

1. Please explain your answer:
2. Do you think that this goal of the Dutch government is achievable in your field? *

- Yes
- No
- I don’t know

1. Please explain your answer:
2. Would you support the goal to be world-leader in animal-free innovations in your own country? *

- Yes
- No
- I don’t know

## S3.3: Roadblocks

1. There could be several reasons why animal-free methods are not implemented in your field of science. Please indicate below how important you think the roadblocks are in your field of science, with 1 as not important and 4 as very important. *


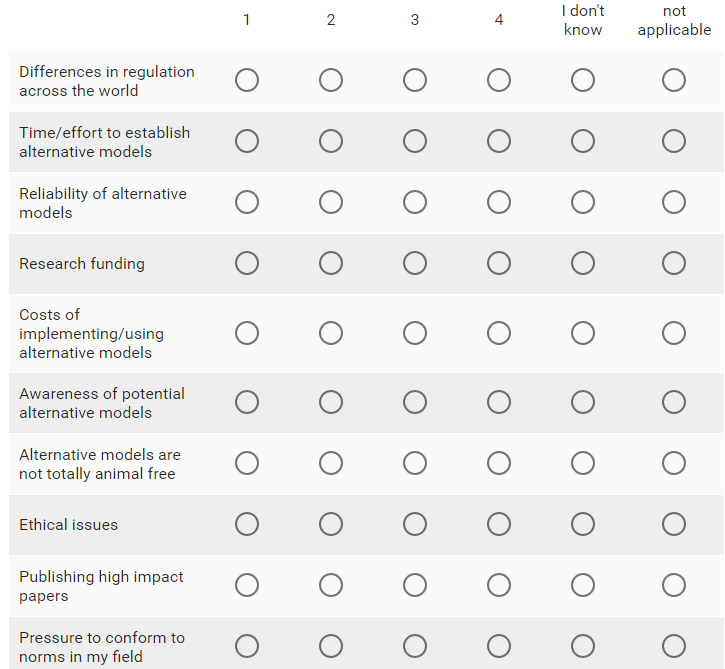


1. If you experience other roadblocks, please write them down below:

## S3.4: Demographics

Thank you for filling in the survey so far. We would like to collect some demographic data to place our research in more perspective. Please note that all data is collected anonymously and that you are able to opt out at any stage of the progression. The questions without an asterisk are optional to fill in.

1. Age
2. Gender

- Male
- Female

1. In which sector are you working? *

- Academia
- Pharmaceutical
- Non-profit
- Government
- Contract Research Organisation
- Other:

1. What is your educational background (for example biology, pharmacology, etc.)?
2. Was any education given on performing animal experiments in your curriculum?

- Yes
- No
- I don’t recall

1. In which scientific field are you currently working?
2. What is your level? *

- PhD
- Postdoc
- Principal investigator
- Director
- Head of the department
- Regular employee
- Student
- Other:

1. What is your nationality?
2. In which country are you currently working?

- Belgium
- The Netherlands
- France
- Germany
- United States of America
- United Kingdom
- India
- Other:

1. Do you working with animals for your research at the moment? *

- Yes (go to question 5.1)
- No (go to question 6.1)

## S3.5: Demographics – details of people working with animals

1. What kind of laboratory animal do you use? *
2. Did you ever consider using alternatives/animal – free methods?

- Yes
- No

1. Please explain what kind of method this would be and why you considered using it:
2. Would you consider moving to another country if the animal research you perform right now is not possible anymore in the country where you work at the moment (for example, due to legislation)? *

- Yes
- No
- Maybe

1. Please explain your answer:

## S3.6: Demographics – details of people working without animals

1. Why do you not use animals in your research? *

- No opportunity
- Not relevant for my research
- Ethical issues
- There are viable non-animal methods
- The costs of using animals are too high

1. What kinds of alternative/animal-free methods do you use for your research? *

- Cell cultures
- Invertebrates
- Computer models
- Humans
- Other:

1. Have you worked with animals in previous experiments? *

- Yes
- No

1. Would you consider using laboratory animals for your research?

- Yes
- No
- Maybe

**References**

1. Brunner D, Frank J, Appl H, Schoffl H, Pfaller W, Gstraunthaler G. Serum-free cell culture: the serum-free media interactive online database. Altex. 2010;27(1):53-62.

2. Altweb. Special section: Monoclonal antibodies [cited 2017 17 April]. Available from: http://altweb.jhsph.edu/mabs/mabs.html.

3. Altweb. Special section: Recombinant antibodies (rAbs) produced without the use of animals [cited 2017 17 April]. Available from: <http://altweb.jhsph.edu/mabs/rabs.html>.

4. Nationaal Kenniscentrum Alternatieven voor dierproeven: Proefdieren in het onderwijs [cited 2017 14 February]. Available from: http://www.nkca.nl/thema-s/proefdieren-in-het-onderwijs/.

5. Openaccess.nl. In the Netherlands [cited 2017 14 Febuary]. Available from: http://openaccess.nl/en/in-the-netherlands.

6. Patil P, Peng RD, Leek J. A statistical definition for reproducibility and replicability. bioRxiv. 2016.

7. Balls M. Replacement of animal procedures: alternatives in research, education and testing. Laboratory animals. 1994;28(3):193-211.

8. Ghalzouri AE. 3V's in de praktijk: Nationaal Kenniscentrum Alternatieven voor dierproeven; [cited 2017 26 February]. Available from: http://www.nkca.nl/3v-s-in-de-praktijk/ontwikkelen/abdoelwaheb-el-ghalzouri-levende-huidmodellen/.

9. Hendriksen C. 3V's in de praktijk: Nationaal Kenniscentrum Alternatieven voor dierproeven; [cited 2017 26 February]. Available from: http://www.nkca.nl/3v-s-in-de-praktijk/pre-validatie/herman-ko%C3%ABter-valideren-is-complex-en-duur/.
